# Supplementary material for: Meta-unstable mRNAs in activated CD8+ T cells are defined by interlinked AU-rich elements and m6A mRNA methylation
Source: Nat Commun. 2026 Jan 22;17:160. doi: 10.1038/s41467-025-67762-w (PMC12827480; doi:10.1038/s41467-025-67762-w)
Supplement: Supplementary file 1 — Supplementary Information [file 41467_2025_67762_MOESM1_ESM.pdf]

Figure S1

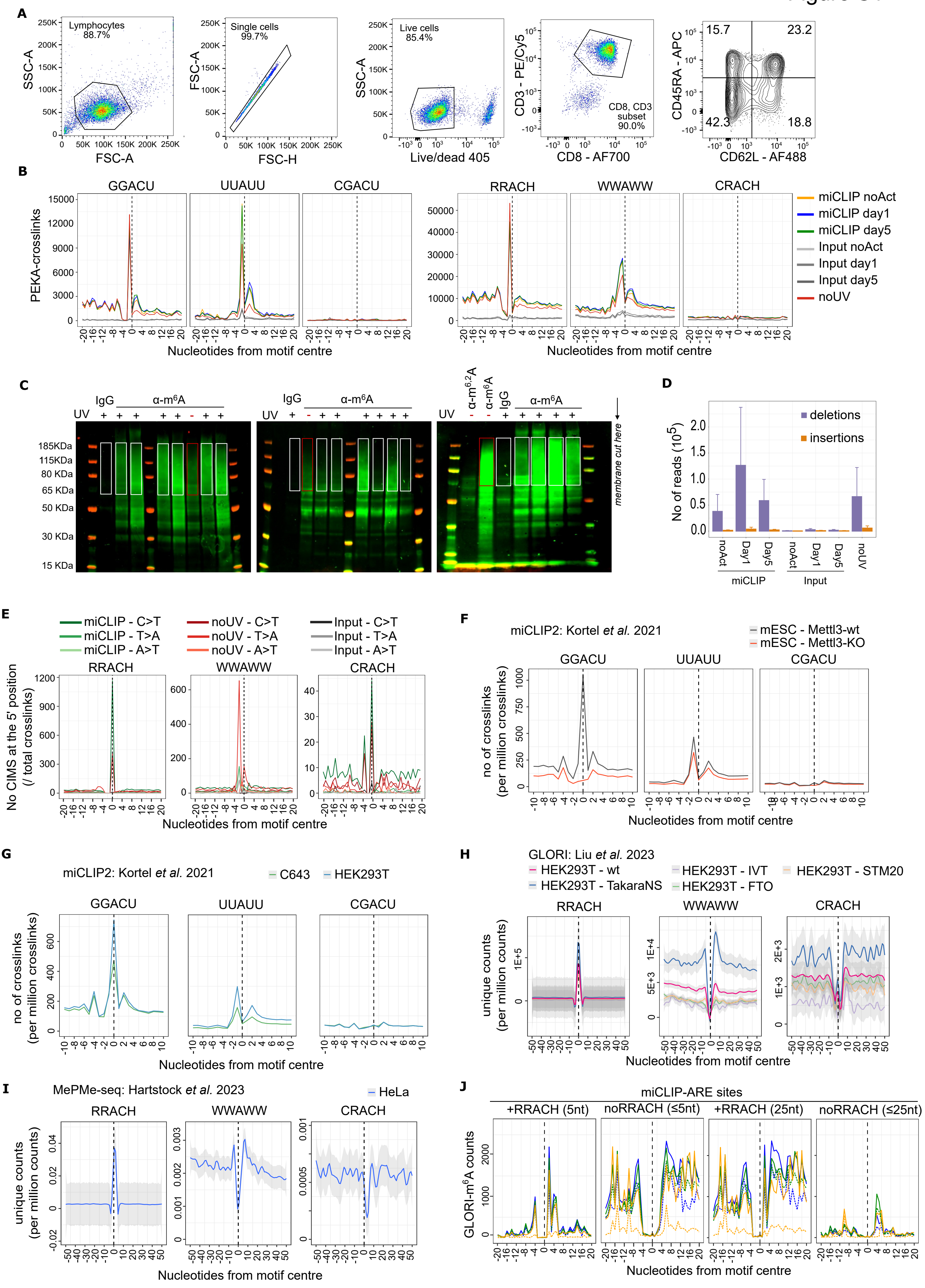
A
Lymphocytes 88.7%
Single cells 99.7%
Live cells 85.4%
CD8, CD3 subset 90.0%
15.7
23.2
42.3
18.8
SSC-A
FSC-A
FSC-H
Live/dead 405
CD3 - PE/Cy5
CD8 - AF700
CD45RA - APC
CD62L - AF488
B
GGACU
UUAUU
CGACU
RRACH
WWAWW
CRACH
PEKA-crosslinks
Nucleotides from motif centre
miCLIP noAct
miCLIP day1
miCLIP day5
Input noAct
Input day1
Input day5
noUV
C
IgG
α-m6A
UV
α-m6,2A
membrane cut here
185KDa
115KDa
80 KDa
65 KDa
50 KDa
30 KDa
15 KDa
D
deletions
insertions
No of reads (10^5)
noAct
Day1
Day5
noUV
miCLIP
Input
E
miCLIP - C>T
miCLIP - T>A
miCLIP - A>T
noUV - C>T
noUV - T>A
noUV - A>T
Input - C>T
Input - T>A
Input - A>T
No CIMS at the 5' position (/ total crosslinks)
F
miCLIP2: Kortel et al. 2021
mESC - Mettl3-wt
mESC - Mettl3-KO
no of crosslinks (per million crosslinks)
G
C643
HEK293T
H
GLORI: Liu et al. 2023
HEK293T - wt
HEK293T - TakaraNS
HEK293T - IVT
HEK293T - FTO
HEK293T - STM20
unique counts (per million counts)
I
MePMe-seq: Hartstock et al. 2023
HeLa
J
miCLIP-ARE sites
+RRACH (5nt)
noRRACH (≤5nt)
+RRACH (25nt)
noRRACH (≤25nt)
GLORI-m6A counts

Figure S2

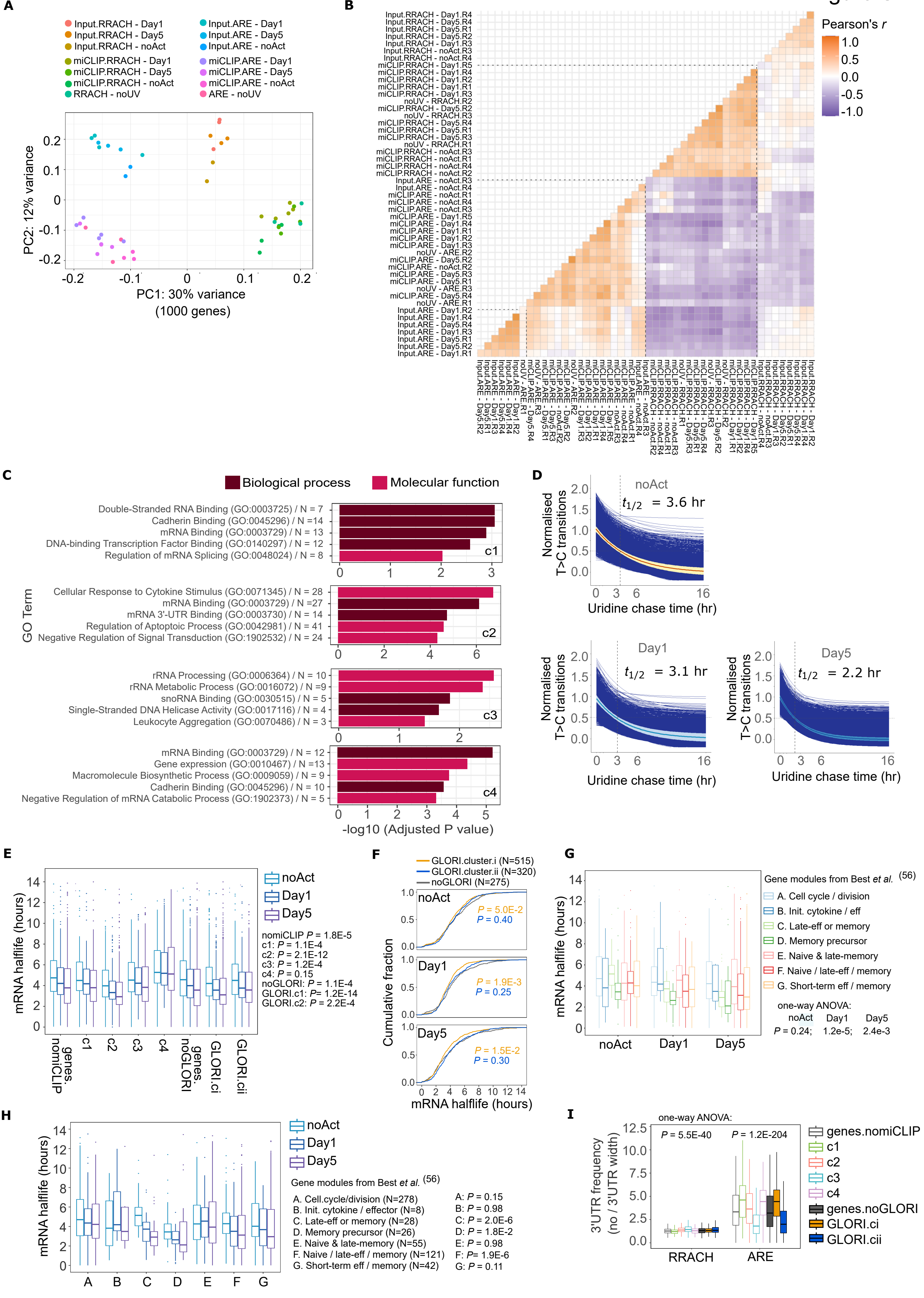
A
Input.RRACH - Day1
Input.RRACH - Day5
Input.RRACH - noAct
miCLIP.RRACH - Day1
miCLIP.RRACH - Day5
miCLIP.RRACH - noAct
RRACH - noUV
Input.ARE - Day1
Input.ARE - Day5
Input.ARE - noAct
miCLIP.ARE - Day1
miCLIP.ARE - Day5
miCLIP.ARE - noAct
ARE - noUV
PC2: 12% variance
PC1: 30% variance
(1000 genes)
B
Pearson's r
1.0
0.5
0.0
-0.5
-1.0
Input.RRACH - Day1.R4
Input.RRACH - Day5.R4
Input.RRACH - Day5.R1
Input.RRACH - Day5.R2
Input.RRACH - Day1.R3
Input.RRACH - noAct.R3
Input.RRACH - noAct.R4
miCLIP.RRACH - Day1.R5
miCLIP.RRACH - Day1.R4
miCLIP.RRACH - Day1.R2
miCLIP.RRACH - Day1.R1
miCLIP.RRACH - Day1.R3
noUV - RRACH.R2
miCLIP.RRACH - Day5.R2
noUV - RRACH.R3
miCLIP.RRACH - Day5.R4
miCLIP.RRACH - Day5.R1
miCLIP.RRACH - Day5.R3
noUV - RRACH.R1
miCLIP.RRACH - noAct.R3
miCLIP.RRACH - noAct.R1
miCLIP.RRACH - noAct.R4
miCLIP.RRACH - noAct.R2
Input.ARE - noAct.R3
Input.ARE - noAct.R4
miCLIP.ARE - noAct.R1
miCLIP.ARE - noAct.R4
miCLIP.ARE - noAct.R3
miCLIP.ARE - Day1.R5
miCLIP.ARE - Day1.R4
miCLIP.ARE - Day1.R1
miCLIP.ARE - Day1.R2
miCLIP.ARE - Day1.R3
noUV - ARE.R2
miCLIP.ARE - Day5.R2
miCLIP.ARE - noAct.R2
miCLIP.ARE - Day5.R3
miCLIP.ARE - Day5.R1
noUV - ARE.R3
miCLIP.ARE - Day5.R4
noUV - ARE.R1
Input.ARE - Day1.R2
Input.ARE - Day1.R4
Input.ARE - Day5.R4
Input.ARE - Day1.R3
Input.ARE - Day5.R1
Input.ARE - Day5.R2
Input.ARE - Day1.R1
C
Biological process
Molecular function
Double-Stranded RNA Binding (GO:0003725) / N = 7
Cadherin Binding (GO:0045296) / N =14
mRNA Binding (GO:0003729) / N = 13
DNA-binding Transcription Factor Binding (GO:0140297) / N = 12
Regulation of mRNA Splicing (GO:0048024) / N = 8
c1
Cellular Response to Cytokine Stimulus (GO:0071345) / N = 28
mRNA Binding (GO:0003729) / N =27
mRNA 3'-UTR Binding (GO:0003730) / N = 14
Regulation of Aptoptoic Process (GO:0042981) / N = 41
Negative Regulation of Signal Transduction (GO:1902532) / N = 24
c2
rRNA Processing (GO:0006364) / N = 10
rRNA Metabolic Process (GO:0016072) / N =9
snoRNA Binding (GO:0030515) / N = 5
Single-Stranded DNA Helicase Activity (GO:0017116) / N = 4
Leukocyte Aggregation (GO:0070486) / N = 3
c3
mRNA Binding (GO:0003729) / N = 12
Gene expression (GO:0010467) / N =13
Macromolecule Biosynthetic Process (GO:0009059) / N = 9
Cadherin Binding (GO:0045296) / N = 10
Negative Regulation of mRNA Catabolic Process (GO:1902373) / N = 5
c4
GO Term
-log10 (Adjusted P value)
D
noAct
t1/2 = 3.6 hr
Day1
t1/2 = 3.1 hr
Day5
t1/2 = 2.2 hr
Normalised T>C transitions
Uridine chase time (hr)
E
noAct
Day1
Day5
mRNA halflife (hours)
nomiCLIP P = 1.8E-5
c1: P = 1.1E-4
c2: P = 2.1E-12
c3: P = 1.2E-4
c4: P = 0.15
noGLORI: P = 1.1E-4
GLORI.c1: P = 1.2E-14
GLORI.c2: P = 2.2E-4
genes.nomiCLIP
c1
c2
c3
c4
genes.noGLORI
GLORI.ci
GLORI.cii
F
GLORI.cluster.i (N=515)
GLORI.cluster.ii (N=320)
noGLORI (N=275)
noAct
P = 5.0E-2
P = 0.40
Day1
P = 1.9E-3
P = 0.25
Day5
P = 1.5E-2
P = 0.30
Cumulative fraction
mRNA halflife (hours)
G
Gene modules from Best et al. (56)
A. Cell cycle / division
B. Init. cytokine / eff
C. Late-eff or memory
D. Memory precursor
E. Naive & late-memory
F. Naive / late-eff / memory
G. Short-term eff / memory
one-way ANOVA:
noAct Day1 Day5
P = 0.24; 1.2e-5; 2.4e-3
mRNA halflife (hours)
noAct
Day1
Day5
H
noAct
Day1
Day5
mRNA halflife (hours)
Gene modules from Best et al. (56)
A. Cell.cycle/division (N=278)
B. Init. cytokine / effector (N=8)
C. Late-eff or memory (N=28)
D. Memory precursor (N=26)
E. Naive & late-memory (N=55)
F. Naive / late-eff / memory (N=121)
G. Short-term eff / memory (N=42)
A: P = 0.15
B: P = 0.98
C: P = 2.0E-6
D: P = 1.8E-2
E: P = 0.98
F: P= 1.9E-6
G: P = 0.11
I
one-way ANOVA:
P = 5.5E-40
P = 1.2E-204
3'UTR frequency (no / 3'UTR width)
RRACH
ARE
genes.nomiCLIP
c1
c2
c3
c4
genes.noGLORI
GLORI.ci
GLORI.cii

Figure S3

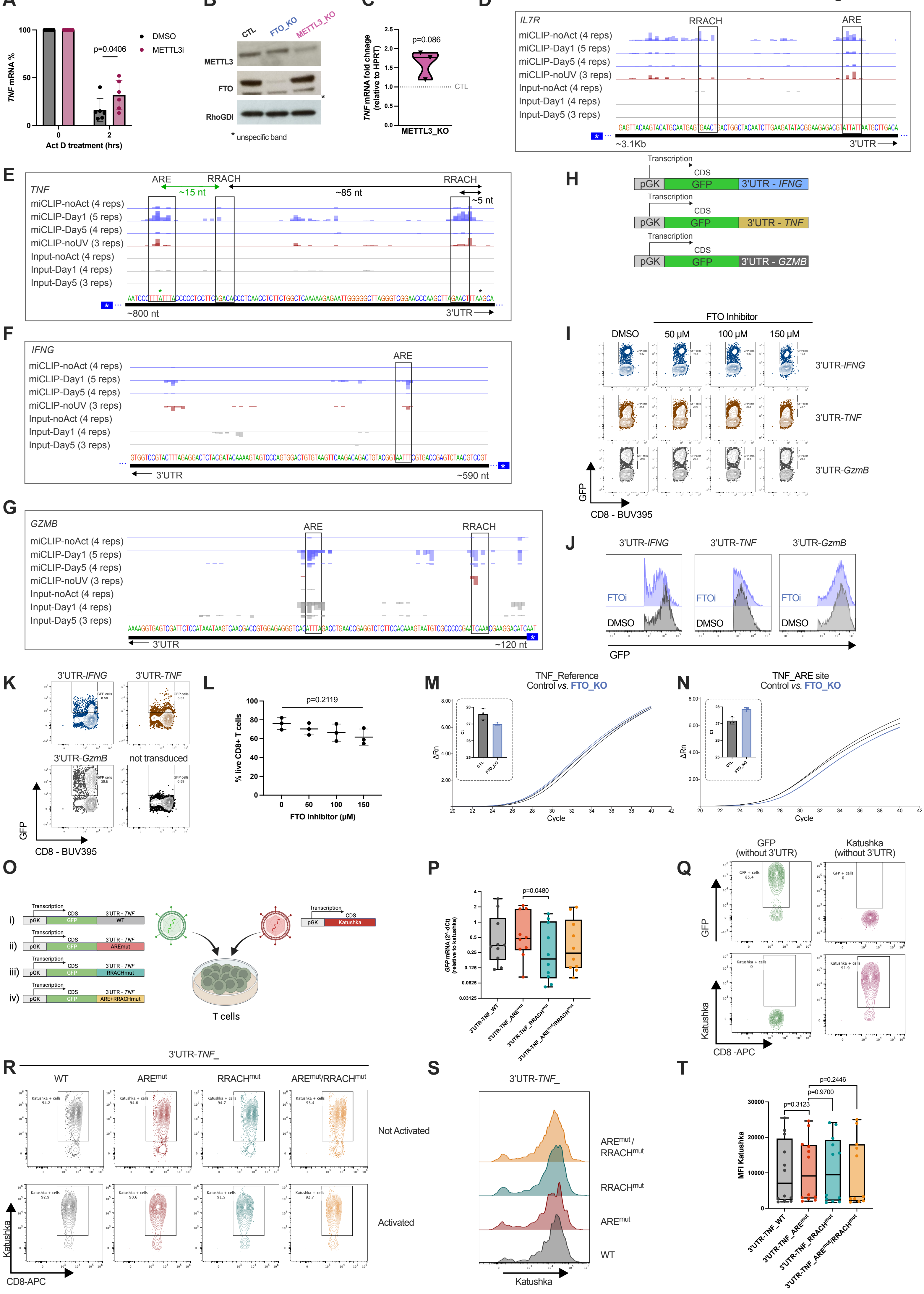
A
TNF mRNA %
Act D treatment (hrs)
p=0.0406
DMSO
METTL3i
B
CTL
FTO_KO
METTL3_KO
METTL3
FTO
RhoGDI
* unspecific band
C
TNF mRNA fold chnage (relative to HPRT)
p=0.086
CTL
METTL3_KO
D
IL7R
RRACH
ARE
miCLIP-noAct (4 reps)
miCLIP-Day1 (5 reps)
miCLIP-Day5 (4 reps)
miCLIP-noUV (3 reps)
Input-noAct (4 reps)
Input-Day1 (4 reps)
Input-Day5 (3 reps)
~3.1Kb
3'UTR
E
TNF
ARE
RRACH
~15 nt
~85 nt
~5 nt
~800 nt
3'UTR
F
IFNG
~590 nt
G
GZMB
~120 nt
H
Transcription
CDS
pGK
GFP
3'UTR - IFNG
3'UTR - TNF
3'UTR - GZMB
I
FTO Inhibitor
DMSO
50 μM
100 μM
150 μM
3'UTR-IFNG
3'UTR-TNF
3'UTR-GzmB
GFP
CD8 - BUV395
J
FTOi
DMSO
GFP
K
not transduced
L
p=0.2119
% live CD8+ T cells
FTO inhibitor (μM)
M
TNF_Reference
Control vs. FTO_KO
ΔRn
Cycle
N
TNF_ARE site
Control vs. FTO_KO
O
3'UTR - TNF
WT
AREmut
RRACHmut
ARE+RRACHmut
Katushka
T cells
P
p=0.0480
GFP mRNA (2^-dCt) (relative to katushka)
3'UTR-TNF_WT
3'UTR-TNF_AREmut
3'UTR-TNF_RRACHmut
3'UTR-TNF_AREmut/RRACHmut
Q
GFP (without 3'UTR)
Katushka (without 3'UTR)
CD8 -APC
R
3'UTR-TNF_
WT
AREmut
RRACHmut
AREmut/RRACHmut
Not Activated
Activated
Katushka
CD8-APC
S
3'UTR-TNF_
AREmut / RRACHmut
RRACHmut
AREmut
WT
Katushka
T
MFI Katushka
p=0.3123
p=0.9700
p=0.2446

Figure S4

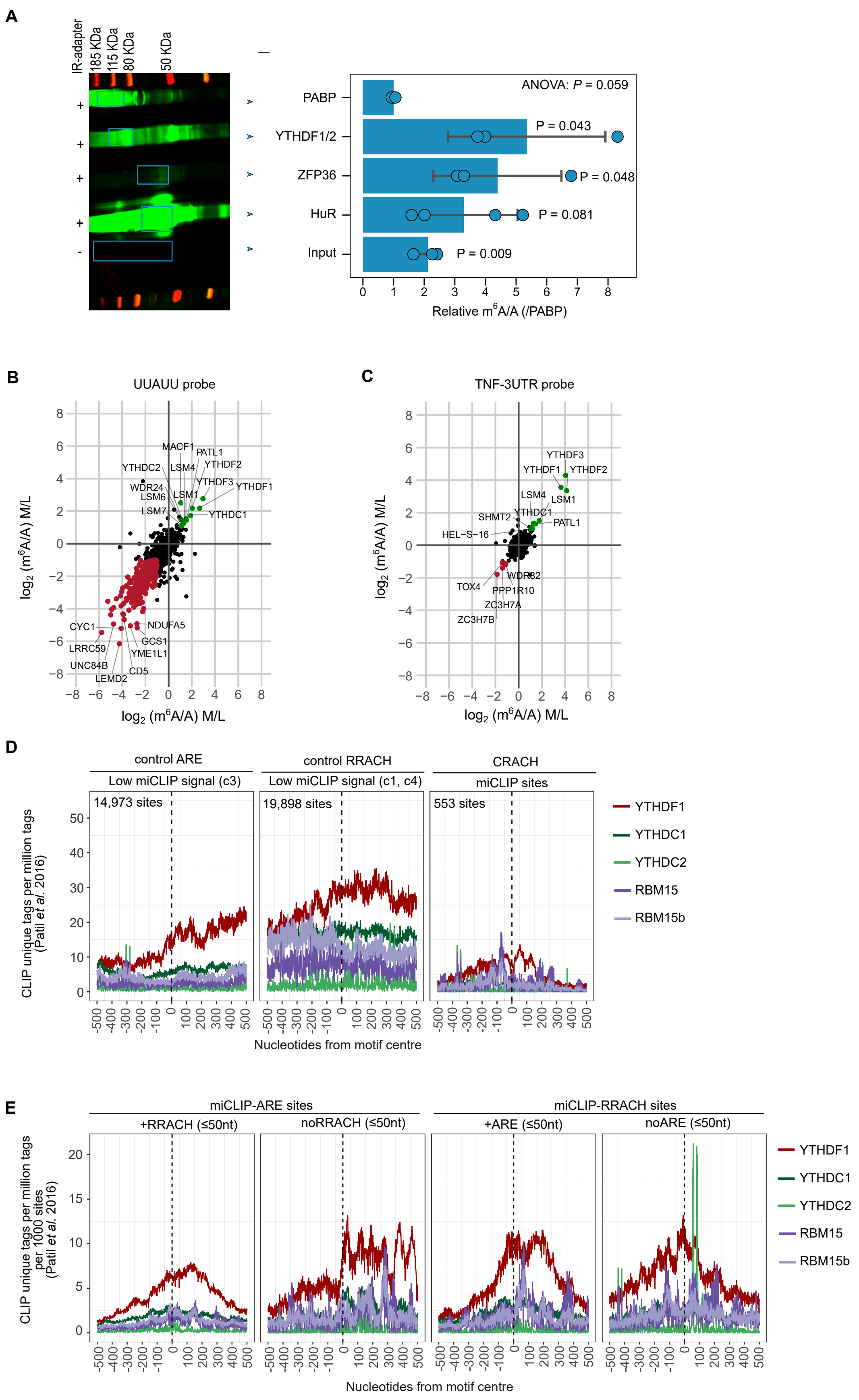

A
IR-adapter
185 KDa
115 KDa
80 KDa
50 KDa
ANOVA: P = 0.059
PABP
YTHDF1/2
ZFP36
HuR
Input
P = 0.043
P = 0.048
P = 0.081
P = 0.009
Relative m6A/A (/PABP)
B
UUAUU probe
log2 (m6A/A) M/L
MACF1
PATL1
YTHDC2
LSM4
YTHDF2
WDR24
LSM6
YTHDF3
YTHDF1
LSM1
LSM7
YTHDC1
CYC1
NDUFA5
GCS1
LRRC59
YME1L1
UNC84B
CD5
LEMD2
C
TNF-3UTR probe
YTHDF3
YTHDF1
YTHDF2
LSM4
LSM1
SHMT2
YTHDC1
PATL1
HEL−S−16
WDR82
TOX4
PPP1R10
ZC3H7A
ZC3H7B
D
control ARE
Low miCLIP signal (c3)
control RRACH
Low miCLIP signal (c1, c4)
CRACH
miCLIP sites
14,973 sites
19,898 sites
553 sites
CLIP unique tags per million tags
(Patil et al. 2016)
Nucleotides from motif centre
YTHDF1
YTHDC1
YTHDC2
RBM15
RBM15b
E
miCLIP-ARE sites
+RRACH (≤50nt)
noRRACH (≤50nt)
miCLIP-RRACH sites
+ARE (≤50nt)
noARE (≤50nt)
CLIP unique tags per million tags
per 1000 sites
(Patil et al. 2016)
Nucleotides from motif centre
YTHDF1
YTHDC1
YTHDC2
RBM15
RBM15b

Figure S1: Motif-specific detection of miCLIP crosslinks and published m^{6}A sequencing methods; related to Figure 1**. (A)** Representative flow cytometry plots of CD8$^+$ T cells isolated from a healthy donor prior to *ex vivo* activation. After magnetic bead isolation, 90% of the cells were CD3- and CD8$^+$ positive. CD62L and CD45RA staining were used to identify the four CD8$^+$ T cell subtypes (T naïve: CD62L+/CD45RA+; T central memory: CD62L+/CD45RA-; T effector memory: CD62L-/CD45RA-; T effector: CD62L-/CD45RA+). **(B)** Metagene distribution of PEKA crosslinks around motif classes (RRACH, WWAWW and CRACH pentamers) and representative motifs of each class (GGACU, UUAUU and CGACU); the number of PEKA crosslinks was averaged across CD8$^+$ T cell replicates. The miCLIP crosslinks located at the center of WWAWW pentamers (5th panel) denote the miCLIP-ARE sites as referred the manuscript. **(C)** Infrared (IR) imaging of the nitrocellulose membrane containing RNA UV-crosslinked (or not; noUV samples) to anti-m^{6}A antibody (miCLIP samples) or to IgG isotype control. The IR signal derives from an IR dye-conjugated adapter ligated to the 3'RNA termini during CLIP. **(D)** Number of deletions and insertions detected by crosslink-induced mutation signatures (CIMS) at cDNA start sites across miCLIP, input and noUV samples; data show mean ± SD. Sample numbers: miCLIP noAct (n=4), Day1 (n=5), Day5 (n=4); Input noAct, Day1 and Day5 (n=12 total; 4 donors each). **(E)** Metagene distribution of CIMS around the RRACH, WWAW and CRACH motif classes. **(F-G)** Distribution of crosslinks around the GGACU, UUAUU and CGACU motifs in mouse embryonic stem cells (F) and human cell lines (G) generated in the miCLIP2 study[29]. **(H-I)** Distribution of m^{6}A sites around the RRACH, WWAWW, and CRACH classes identified in HEK293T (H) and HeLa (I) cells by antibody-free methods; dataset from the GLORI[50] (H) and MePME-Seq (I) studies[51]. **(J)** Metagene distribution of GLORI-determined m^{6}A counts centred on miCLIP-ARE sites that contain nearby RRACH motifs (left) or lack RRACH motifs (right) within the indicated distances, showing the effect of the METTL3 inhibitor. The WWAWW+RRACH sites refer to miCLIP-ARE sites containing at least one RRACH motif within the indicated distance. Source data are provided as a Source Data file.

Figure S2: Dynamics and RNA stability effects of m^{6}A-modified RRACH and ARE motifs in CD8$^+$ T cells; related to Figure 2. **(A)** Projection of DESeq2-normalized miCLIP crosslinks and input counts showing the dissimilarity between miCLIP and input samples in a motif-dependent manner. **(B)** Spearman's correlation grid showing that miCLIP-ARE crosslinks are inversely related (Pearson's $r < 0$) to miCLIP-RRACH crosslinks and weakly correlated (Pearson's $r \sim 0$) with input counts. **(C)** Gene Ontology (GO) enrichment of the gene clusters obtained from the hierarchical clustering of DESeq2-normalized miCLIP and input counts. **(D)** Normalized 4SU content of mRNAs, as determined using the SLAM-seq method, showing a median mRNA half-life ($t_{1/2}$) of 3.6 h in non-activated (noAct), 3.1 h in Day1-activated and 2.2 h in Day5-activated CD8$^+$ T cells (n=2 donors). **(E)** Distribution of mRNA half-lives showing the destabilization of miCLIP and GLORI clusters determined by the DESeq2 model. Statistical comparisons were performed using two-sided Kolmogorov–Smirnov (KS) tests. **(F)** Cumulative distribution of mRNAs with distinct GLORI-m^{6}A signatures in the DESeq2 model, related to Figure 2E. Two-sided KS tests were used for all comparisons. **(G-H)** Distribution of mRNA half-lives across CD8$^+$ gene module at each CD8$^+$ T cell state[57] (G) showing their destabilization upon CD8$^+$ T cell activation (H); two-sided KS tests were used for comparisons. **(I)** Frequencies of RRACH and WWAWW pentamers in the 3'UTRs of DESeq2-cluster mRNAs. Source data are provided as a Source Data file.

Figure S3: Validation and reporter assays targeting miCLIP sites in the *TNF* 3'UTR of CD8$^+$ T cells; related to Figure 3. **(A)** Actinomycin D experiments showing % of remaining mRNA in CD8$^+$ T cells treated with DMSO or METTL3inhibitor. Mean±SD; n=6; paired two-tailed t-test. **(B)** Western blot of FTO and METTL3 knockouts (KO). Representative of n=3. **(C)** *TNF* mRNA levels in METTL3-KO CD8$^+$ T cells in resting state. Fold change relative to DMSO (grey line). Reference gene HPRT. Truncated violin plot, with median; n=3; paired two-way t-tests on ΔCt values. **(D-G)** IGV plots of miCLIP thresholded sites (purple), noUV (red), input (grey) and cDNA start positions of *IL7R* (D), *TNF* (E), *IFNG* (F) and *GZMB* (G) 3'UTR. **(H)** Schematic representation of GFP constructs fused to 3'UTR-*IFNG*, 3'UTR-*TNF* or 3'UTR-*GzmB*. **(I-K)** Representative plots of CD8$^+$ T cells transduced with GFP constructs (S3H); n=3. Cells treated with DMSO or FTOinhibitor and activated with PMA/ionomycin (I). Representative histograms of GFP for DMSO or FTOinhibitor (150µM) (J). Not activated cells - gating strategy is shown (K). **(L)** Percentages of live CD8$^+$ T cells treated with FTOinhibitor Mean±SD; n=3. Ordinary one-way ANOVA and Dunnett's multiple comparisons test. **(M-N)** Representative amplification curves (ΔRn) and Ct quantification for reference (M) and miCLIP-ARE (N) amplicons in control (grey) and FTO-KO (purple) samples. Mean±SD; n=3. **(O)** CD8$^+$ T cells retrovirally transduced with GFP constructs fused to 3'UTR-*TNF*_WT, 3'UTR-*TNF*_ARE^{mut}, 3'UTR-*TNF*_RRACH^{mut} or 3'UTR-*TNF*_ARE^{mut}/RRACH^{mut}. Cells were simultaneously transduced with Katushka-no_3'UTR for transduction reference. Created in BioRender. Foskolou,I. (2026) https:// BioRender.com/5zdrqye. **(P)** GFP mRNA levels (2^-ΔCt) of CD8$^+$ T cells transduced with the GFP constructs (S3O); n=10. RM one-way ANOVA and Tukey's multiple comparisons test on ΔCt values. Box-and-whisker plots (min–max, median, 25th–75th percentiles). **(Q)** Representative plots of CD8$^+$ T cells transduced with GFP-no_3'UTR or Katushka-no_3'UTR constructs. **(R-T)** Katushka protein levels in CD8$^+$ T cells transduced with the constructs of (S3O). Representative plots of resting or PMA/ionomycin-activated cells (R). Katushka MFI quantification in activated cells (S–T) (n=12); paired two-tailed t-tests. Box-and-whisker plots (min–max, median, 25th–75th percentiles). All n values reflect distinct donors. Source data are provided as a Source Data file.

Figure S4: Molecular effects of the miCLIP-ARE site on RNA-binding properties; related to Figure 4. **(A)** CLIP-MS workflow in Jurkat T cells. Left: SDS–PAGE showing RNA-protein complexes purified either from total cell lysate (input) or following RBP immunoprecipitation. Right: LC–MS/MS quantification of m^6A:A ratios in recovered RNAs, normalised to polyA$^+$-binding protein (PABP). Data shown as mean +/- SD. Sample numbers: PABP (n=3), YTHDF1/2 (n=3), ZFP (n=3), HuR (n=4), Input (n=3). **(B-C)** Replicate RNA-pulldown proteomics experiments in human T cells (n = 9 pooled donors). Shown are $\log_2$ fold-changes in RBP abundance for methylated versus unmethylated ARE probes (B) and TNF-3′UTR probes (C). **(D)** Metagene distribution of CLIP data for m^6A-reader proteins[68] at expression-matched control sites in the 3′UTR: ARE sites (left), RRACH sites (middle) and CRACH sites (right). Control sites were selected from DESeq2-defined clusters with low miCLIP signal (cluster 3 for AREs; clusters 1 and 4 for RRACHs). **(E)** Metagene distribution corresponding to Figure 4D, showing CLIP signal at composite versus isolated miCLIP-ARE sites (left two panels) and composite versus isolated miCLIP-RRACH sites (right two panels). Here, CLIP counts are additionally normalised to the total number of sites in each category, enabling direct comparison of signal magnitude across motif classes. Source data are provided as a Source Data file.

Supplementary Data 1: Summary of miCLIP, GLORI, and RNA stability datasets. Sheet 1: DESeq2-normalised miCLIP (PEKA crosslink) counts for 3′UTRs across all $CD8^+$ T-cell activation states, used for clustering analyses in Figures 2 and S2. Sheet 2: DESeq2-normalised GLORI m^6A counts at RRACH and RRACH-flanking-ARE sites, used to define GLORI clusters and assess m^6A dynamics in Figures 2 and S2. Sheet 3: mRNA half-life values (SLAM-seq) for each $CD8^+$ T-cell state (noAct, Day1, Day5), including their assignment to miCLIP/GLORI DESeq2 clusters and $CD8^+$ gene modules. Sheet 4: Gene-level overlap between miCLIP-defined and GLORI-defined clusters, used to compare motif- and method-specific m^6A signatures.

Supplementary Methods

Key Resources Table

REAGENT or RESOURCE	SOURCE	IDENTIFIER (RRID)
**Antibodies**		
CD45RA (human); Flow cytometry	Biolegend (Clone HI100; Cat. No. 304112)	AB_314416
CD62L (human); Flow cytometry	Biolegend (Clone DREG-56; Cat. No. 304816)	AB_528857
CD3 (human); Flow cytometry	Biolegend (Clone HIT3a; Cat. No. 300309)	AB_314045
CD4 (human); Flow cytometry	Biolegend (Clone A161A1; Cat. No. 357418)	AB_2616933
CD8$^+$a (human); Flow cytometry	BD Biosciences (Clone SK1; Cat. No. 563919, 612889, 345775, 570814)	AB_2722546<br>AB_2833078<br>AB_3686054
CD8$^+$a (human); Flow cytometry	Biolegend (Clone SK1; Cat. No. 344710)	AB_2044010
CD8$^+$a (human); Flow cytometry	Biolegend (Clone HIT8a; Cat. No. 300919)	AB_528884
CD127 (IL7Rα); Flow cytometry	BD Biosciences (Clone HIL-7R-M21; Cat. No. 742547)	AB_2740857
anti-mouse IgG2a	Biolegend (Cat. No. 407102)	AB_345322
Dynabeads human T-activator CD3/CD28	Gibco (Cat. No. 11132D)	AB_2943359
Anti-m6A Antibody; miCLIP	Abcam (Cat. No. ab151230)	AB_2753144
Anti-human METTL3; Western Blot	proteintech (Cat. No. 15073-1-AP)	AB_2142033
Anti-human FTO; Western Blot	Abcam (Cat. No. ab92821)	AB_10565042
Mouse IgG HRP-conjugated; Western Blot	R&D (Cat. No. HAF007)	AB_357234
Rabbit IgG HRP-conjugated; Western Blot	R&D (Cat. No. HAF008)	AB_357235
Anti-human RhoGDI; Western Blot	Abnova (Cat. No. 89-113-917)	n/a
FcR Block human	MACS Miltenyi (Cat. No. 120-000-826)	AB_2892112
CD8$^+$ MicroBeads (human)	MACS Miltenyi (Cat. No. 130-045-201)	AB_2889920
Total CD8$^+$ T cell isolation kit (human)	MACS Miltenyi (Cat. No. 130-096-495)	AB_3073903
Anti-human CD28	Biolegend (Clone CD28.2; Cat. No. 302902)	AB_314304
Anti-human YTHDF1	Proteintech (Cat. No. 17479-1-AP)	AB_2217473
Anti-human YTHDF2	Aviva Systems Biology (Cat. No. ARP67917_P050)	AB_2861185
Anti-human HuR	Santa Cruz Biotechnology (Cat. No. SC-5261	AB_627770
Anti-human PABP	Thermo Fisher Scientific (Cat. No. PA5-66900)	AB_2662551
Anti-human ZFP36	Proteintech (Cat. No. 12306-1-AP),	AB_2737443,

	Cell Signaling Technology (Cat. No. 71632)	AB_2799806
anti-human CD3	Biolegend (Clone HIT3a; Cat. No. 300302)	AB_11150396
**Bacterial and virus strains**		
Subcloning Efficiency DH5a Competent Cells	Invitrogen	18265017
**Biological samples**		
Peripheral blood mononuclear cells (PBMCs) were obtained from healthy donors with consent	Cambridge Bioscience; National Health Service (NHS) Blood and Transplant (NHSBT: Addenbrooke's Hospital, Cambridge, UK)	n/a
Peripheral blood mononuclear cells (PBMCs) were obtained from healthy donors with consent	Sanquin Blood Bank (NL)	n/a
**Chemicals, peptides, and recombinant proteins**		
Actinomycin D	Sigma-Aldrich	A9415
BD CellFIX	BD Biosciences	340181
Brefeldin A	Invitrogen	00-4506-51
cOmplete^{TM}, EDTA-free Protease Inhibitor Cocktail	Roche	11836170001
Ficoll-Paque PLUS density gradient separation	GE Healthcare	71-7167-00 AF
FTO inhibitor	Cayman Chemical	70550
GeneJammer	Agilent	204130
Human IL-2 (recombinant)	Roche	1101 1456 001
Human IL-2 (recombinant; Proleukin, Aldesleukin)	Clinigen	n/a
Ionomycin calcium salt	Sigma–Aldrich	13909
Live/Dead Fixable Viability Dyes	Invitrogen	L34963, L34957 & L10119
METTL3 inhibitor	MedChemExpress	HY-134836
OneComp eBeads	Invitrogen	01-1111-42
Phorbol 12-myristate 13-acetate (PMA)	Sigma–Aldrich	P8139
Power SYBR^{TM} Green PCR Master Mix	Applied Biosystems	4367660
RetroNectin	Takara Bio	T100B
RNase A, DNase and protease-free	Thermo Fisher Scientific	EN0531
RNaseOUT^{TM} Recombinant Ribonuclease Inhibitor	Invitrogen	10777019
TRIzol Reagent	Invitrogen	15596018

TrueCut™ Cas9 Protein v2	Invitrogen	A36499
rCutSmart™ Buffer	New England BioLabs	B6004S
Bst 2.0 DNA polymerase	New England BioLabs	M0537S
SplintR Ligase	New England BioLabs	M0375S
ATP Solution (10 mM)	Thermo Fisher Scientific	PV3227
dTTP (10 mM)	Thermo Fisher Scientific	18255018
Pierce™ ECL Western Blotting Substrate	Invitrogen	32106
**Critical commercial assays**		
Cytofix/Cytoperm Fixation/Permeabilization kit	BD Biosciences	554714
iScript™ cDNA Synthesis Kit	Bio-Rad	1708890
P2 Primary Cell 4D-Nucleofector™ X Kit L	Lonza	V4XP-2024
True-Nuclear Transcription Factor Buffer Set	Biolegend	424401
**Experimental models: Cell lines**		
FLYRD18 cells	ECACC	95091902
Jurkat T cells (JH 6.2 line)	Cell Services - Francis Crick Institute	JH 6.2
**Oligonucleotides**		
Probes for RNA pulldowns with dimethyl labelling		
RRACH control probe[75]	CCACGACAAGAGACGGACUCCCUCGAGAGACAGUUAA	Custom made
RRACH m^6A probe[75]	CCACGACAAGAGACGG**m^6A**CUCCCUCGAGAGACAGUUAA	Custom made
ARE control probe	CCACGACAAGAGACUUAUUCCCUCGAGAGACAGUUAA	Custom made (this work)
ARE m^6A probe	CCACGACAAGAGACUU**m^6A**UUCCCUCGAGAGACAGUUAA	Custom made (this work)
TNFα control probe	UGCCCCAAUCCCUUUAUUACCCCCUCCUUCAGACA	Custom made (this work)
TNFα_m^6A probe	UGCCCCAAUCCCUUU**m^6A**UUACCCCCUCCUUCAGACA	Custom made (this work)
SELECT primers		
TNF_ARE_up	tagccagtaccgtagtgcgtgGTCTGAAGGAGGGGGTAA	Custom made (this work)
TNF_ARE_down	5phos/AAAGGGATTGGGGCAGGGcagaggctgagtcgctgcat	Custom made (this work)
TNF_REF_up	tagccagtaccgtagtgcgtgTTTCGAAGTGGTGGTCTTGTTGC	Custom made (this work)

TNF_ REF _down	5phos/TAAAGTTCTAAGCTTGGGTTCCG cagaggctgagtcgctgcat	Custom made (this work)
qPCR primers		
qPCR_F for SELECT[65]	5’-ATGCAGCGACTCAGCCTCTG-3’	Custom made
qPCR_R for SELECT[65]	5’-TAGCCAGTACCGTAGTGCGTG-3’	Custom made
TNFa Fw[76]	5’-GCCAGAGGGCTGATTAGAG-3’	Custom made
TNFa Rev[76]	5’-TCAGCCTCTTCTCCTTCCTG-3’	Custom made
IFNG Fw[77]	5’-AGCTCTGCATCGTTTTGGGTT-3’	Custom made
IFNG Rev[77]	5’-GTTCCATTATCCGCTACATCTGAA-3’	Custom made
ZFP36L1 Fw[78]	5’-CCCGACCTTGGACAACTCAA-3’	Custom made
ZFP36L1 Rev[78]	5’-CTGCAGACCCTGGCTTAGTC-3’	Custom made
HPRT Fw[79]	5’-TGACACTGGCAAAACAATGCA-3’	Custom made
HPRT Rev[79]	5’-GGTCCTTTTCACCAGCAAGCT-3’	Custom made
18S Fw[76]	5’-AGACAACAAGCTCCGTGAAGA-3’	Custom made
18S Rev[76]	5’-CAGAAGTGACGCAGCCCTCTA-3’	Custom made
GFP_Fw	5’-ATCCTGGGGCACAAGCTGGAGT-3’	Custom made (this work)
GFP_Rev	5’-TTCTGCTGGTAGTGGTCGGCGA-3’	Custom made (this work)
Katushka_Fw	5’-TGACCGCTACCCAGGACACCAG-3’	Custom made (this work)
Katushka_Rev	5’-GGCCTCCCAGCCGAGTGTTTTC-3’	Custom made (this work)
IL7R Fw	5’-TCGCAGCACTCACTGACCTGTG-3’	Custom made (this work)
IL7R Rev	5’-CCTCCACGAGGGCCCCACATAT-3’	Custom made (this work)
CCL4 Fw	5’-AATGGGCTCAGACCCTCCCACC-3’	Custom made (this work)
CCL4 Rev	5’-ATACCACAGCTGGCTGGGAGCA-3’	Custom made (this work)
CD69 Fw	5’-ACCTGGTCACCCATGGAAGTGGT-3’	Custom made (this work)
CD69 Rev	5’-TCCATGCTGCTGACCTCTGTGT-3’	Custom made (this work)
CD28 Fw	5’-GGTTGGTGGAGTCCTGGCTTGC-3’	Custom made (this work)
CD28 Rev	5’-GCGGCGGGGAGTCATGTTCATG-3’	Custom made (this work)

TCF7 Fw	5'-TGCAGCTATACCCAGGCTGG-3'	Custom made (this work)
TCF7 Rev	5'-CCTCGACCGCCTCTTCTTC-3'	Custom made (this work)
CRISPR-Cas9 crRNAs		
Human METTL3[80]	5'-GGACACGTGGAGCTCTATCC-3′	Custom made
Human FTO	5'-GTCTAATATAAAACACACCG-3′	Custom made (this work)
Alt-R® CRISPR-Cas9 Negative Control crRNA #1	IDT	1072544
CRISPR-Cas9 tracrRNA	IDT	1072533
**Recombinant DNA**		
pRETRO-SUPER GFP	Brummelkamp *et al.,* 2002	n/a
pMIG-w plasmid	Addgene	12282
**Software and algorithms**		
Flow bio	https://app.flow.bio/	n/a
FlowJo v10	BD Biosciences (https://www.flowjo.com/)	n/a
Design & Analysis 2 (DA2) Software v2.6.0	Thermo Fisher Scientific	n/a
GraphPad Prism v10	GraphPad Software (https://www.graphpad.com/)	n/a
R-4.3.1	https://cran.r-project.org/bin/windows/base/old/4.3.1/	n/a
Skyline (version 19.1)	https://skyline.ms/project/home/software/Skyline/begin.view	n/a
**Other**		
Fetal Bovine Serum (FBS)	Sigma	F7524
Penicillin and Streptomycin	Sigma	P0781
RPMI media	Gibco	52400-025
DMEM	Thermo Fisher Scientific	11995065
4D-Necleofector X Unit	Lonza	Cat. No. AAF-1003X
