# Supplementary material for: Meta-unstable mRNAs in activated CD8+ T cells are defined by interlinked AU-rich elements and m6A mRNA methylation
Source: Nat Commun. 2026 Jan 22;17:160. doi: 10.1038/s41467-025-67762-w (PMC12827480; doi:10.1038/s41467-025-67762-w)
Supplement: Supplementary file 2 — Description of Additional Supplementary Files [file 41467_2025_67762_MOESM2_ESM.pdf]

Description of Additional Supplementary Files

Supplementary Data 1: Summary of miCLIP, GLORI, and RNA stability datasets.

Sheet 1: DESeq2-normalised miCLIP (PEKA crosslink) counts for 3′UTRs across all $CD8^+$ T-cell activation states, used for clustering analyses in Figures 2 and S2.
Sheet 2: DESeq2-normalised GLORI m^6A counts at RRACH and RRACH-flanking-ARE sites, used to define GLORI clusters and assess m^6A dynamics in Figures 2 and S2.
Sheet 3: mRNA half-life values (SLAM-seq) for each $CD8^+$ T-cell state (noAct, Day1, Day5), including their assignment to miCLIP/GLORI DESeq2 clusters and $CD8^+$ gene modules.
Sheet 4: Gene-level overlap between miCLIP-defined and GLORI-defined clusters, used to compare motif- and method-specific m^6A signatures.
