# Supplementary material for: Meta-unstable mRNAs in activated CD8+ T cells are defined by interlinked AU-rich elements and m6A mRNA methylation
Source: Nat Commun. 2026 Jan 22;17:160. doi: 10.1038/s41467-025-67762-w (PMC12827480; doi:10.1038/s41467-025-67762-w)
Supplement: Supplementary file 4 — Reporting Summary [file 41467_2025_67762_MOESM4_ESM.pdf]

nature portfolio

Corresponding author(s): Jernej Ule; Randall S. Johnson

Last updated by author(s): Oct 7, 2025

Reporting Summary

Nature Portfolio wishes to improve the reproducibility of the work that we publish. This form provides structure for consistency and transparency in reporting. For further information on Nature Portfolio policies, see our Editorial Policies and the Editorial Policy Checklist.

Statistics

For all statistical analyses, confirm that the following items are present in the figure legend, table legend, main text, or Methods section.

n/a	Confirmed	
☐	☒	The exact sample size (*n*) for each experimental group/condition, given as a discrete number and unit of measurement
☐	☒	A statement on whether measurements were taken from distinct samples or whether the same sample was measured repeatedly
☐	☒	The statistical test(s) used AND whether they are one- or two-sided<br>*Only common tests should be described solely by name; describe more complex techniques in the Methods section.*
☐	☒	A description of all covariates tested
☐	☒	A description of any assumptions or corrections, such as tests of normality and adjustment for multiple comparisons
☐	☒	A full description of the statistical parameters including central tendency (e.g. means) or other basic estimates (e.g. regression coefficient) AND variation (e.g. standard deviation) or associated estimates of uncertainty (e.g. confidence intervals)
☐	☒	For null hypothesis testing, the test statistic (e.g. *F*, *t*, *r*) with confidence intervals, effect sizes, degrees of freedom and *P* value noted<br>*Give P values as exact values whenever suitable.*
☒	☐	For Bayesian analysis, information on the choice of priors and Markov chain Monte Carlo settings
☐	☒	For hierarchical and complex designs, identification of the appropriate level for tests and full reporting of outcomes
☐	☒	Estimates of effect sizes (e.g. Cohen's *d*, Pearson's *r*), indicating how they were calculated

Our web collection on statistics for biologists contains articles on many of the points above.

Software and code

Policy information about availability of computer code

Data collection	Next-generation sequencing data were generated on Illumina platforms according to the manufacturer’s protocols. Mass spectrometry data were acquired on TSQ Quantiva Thermo Scientific instruments using vendor-recommended acquisition settings and optimized at MS facilities. Flow cytometry data were collected using BD FACSDiva software (v9.0) and Sony Spectra Analyser software and analyzed using FlowJo (BD Biosciences, v10). Raw data files were exported in standard formats for downstream analysis.
Data analysis	Microsoft Excel for Microsoft 365 MSO (Version 2507 Build 16.0.19029.20136) 64-bit<br>GraphPad Prism Version 10.4.1 (532)<br>Plots were generated using R version 4.5.1 (2025-06-13) and Graphpad Prism.<br>Skyline version 19.1 (MacCoss Lab Software).

For manuscripts utilizing custom algorithms or software that are central to the research but not yet described in published literature, software must be made available to editors and reviewers. We strongly encourage code deposition in a community repository (e.g. GitHub). See the Nature Portfolio guidelines for submitting code & software for further information.

Data

Policy information about availability of data

All manuscripts must include a data availability statement. This statement should provide the following information, where applicable:

- Accession codes, unique identifiers, or web links for publicly available datasets
- A description of any restrictions on data availability
- For clinical datasets or third party data, please ensure that the statement adheres to our policy

All datasets generated in this study are publicly available, as described in the manuscript and below:

• miCLIP - sequencing data ArrayExpress (E-MTAB-15643); sequence raw and processed files: https://app.flow.bio/projects/663588932565340992/
• GLORI - sequencing data ArrayExpress (E-MTAB-15649); sequence raw and processed files: https://app.flow.bio/projects/127418738102891967/
• SLAM-Seq - sequencing data ArrayExpress (E-MTAB-15648); sequence raw and processed files: https://app.flow.bio/projects/783560763419435859/ (SLAM-Seq).
• The mass spectrometry proteomics data have been deposited to the ProteomeXchange Consortium via the PRIDE92 partner repository with the dataset identifier PXD059083.
• Flow cytometry, western blots and analysed data are available at Mendeley Data: Mendeley Data, V1, doi: 10.17632/bhgn4bn5ks.1
• Source data are provided with this paper.

Research involving human participants, their data, or biological material

Policy information about studies with human participants or human data. See also policy information about sex, gender (identity/presentation), and sexual orientation and race, ethnicity and racism.

Reporting on sex and gender	No selection based on sex or gender was applied, as the study focused on primary human CD8⁺ T cell biology and did not anticipate sex-specific differences in the measured outcomes.
Reporting on race, ethnicity, or other socially relevant groupings	No selection based on race, ethnicity, or other socially relevant characteristics was applied; these variables were not expected to influence the study's molecular endpoints.
Population characteristics	Participants were healthy adult blood donors; no other specific population characteristics were selected to avoid bias in immune cell function.
Recruitment	Donors were recruited through the NHSBT Blood Donor system and Sanquin Bloed Bank without targeted selection, ensuring a representative volunteer pool within standard donor eligibility criteria.
Ethics oversight	The study was performed according to the Declaration of Helsinki (seventh revision, 2013). Ethical approval was obtained from the Eastern England-Cambridge Central Research Ethics Committee (06/Q0108/281), and consent was obtained from all the subjects. Written informed consent was obtained (Cambridge Bioscience, Cambridge, UK; NHSBT Cambridge, UK; Sanquin Research, Amsterdam, NL).

Note that full information on the approval of the study protocol must also be provided in the manuscript.

Field-specific reporting

Please select the one below that is the best fit for your research. If you are not sure, read the appropriate sections before making your selection.

☒ Life sciences ☐ Behavioural & social sciences ☐ Ecological, evolutionary & environmental sciences

For a reference copy of the document with all sections, see nature.com/documents/nr-reporting-summary-flat.pdf

Life sciences study design

All studies must disclose on these points even when the disclosure is negative.

Sample size	Figures 1–4 and S1–S4: Three or more biological replicates were used unless otherwise stated.<br>-miCLIP and Input samples: Four biological replicates of primary CD8⁺ T cells were collected for each activation state (noAct, Day 1, and Day 5), except for miCLIP–Day 1 samples, which had five replicates.<br>-GLORI samples: Two biological replicates of primary CD8⁺ T cells per activation state (noAct, Day 1, and Day 5) were analysed, including two replicates treated with a METTL3 inhibitor and two with DMSO control.<br>-SLAM-Seq samples: Two biological replicates of primary CD8⁺ T cells per activation state (noAct, Day 1, and Day 5) were collected at each time point (0 h, 1 h, 3 h, 6 h, and 16 h).<br>-MS proteomics: Two biological replicates of activated CD8⁺ T cells were analysed, with each replicate representing a pooled sample from nine donors.
Data exclusions	Four Input replicates were excluded from the DESeq2 model in Figure 2A (noAct, replicates 1 and 2; Day 1, replicate 1; Day 5, replicate 3) due to low read counts (<500) in the 3′UTR.

	Two donors were excluded from Supplemental Fig. 3T due to limited number of positive cells (transduction efficience <5%), which did not allow for reliable MFI values.
Replication	All key experimental findings were confirmed by the stated number of biological replicates. All replication attempts were successful, and results were reproducible.
Randomization	Sample allocation was not randomized. To minimize batch effects, replicates in miCLIP, GLORI, and SLAM-Seq experiments were multiplexed during library preparation. When applicable, miCLIP and corresponding Input libraries were prepared in parallel but processed separately.
Blinding	Samples were assigned numerical codes where feasible. However, complete blinding was not possible, as cell culture, sample preparation, library generation, sequencing multiplexing, MS acquisition, and data analysis were often performed by the same operator.

Reporting for specific materials, systems and methods

We require information from authors about some types of materials, experimental systems and methods used in many studies. Here, indicate whether each material, system or method listed is relevant to your study. If you are not sure if a list item applies to your research, read the appropriate section before selecting a response.

Materials & experimental systems

n/a	Involved in the study
☐	☒ Antibodies
☐	☒ Eukaryotic cell lines
☒	☐ Palaeontology and archaeology
☒	☐ Animals and other organisms
☒	☐ Clinical data
☒	☐ Dual use research of concern
☒	☐ Plants

Methods

n/a	Involved in the study
☒	☐ ChIP-seq
☐	☒ Flow cytometry
☒	☐ MRI-based neuroimaging

Antibodies

Antibodies used	All antibodies, including clone, supplier and catalogue number, are listed in the Methods section. Antibodies were used for flow cytometry, immunoblotting, and immunoprecipitation according to manufacturer-recommended dilutions, unless otherwise specified.<br><br>Key antibodies include:<br>• Anti-CD45RA: Biolegend (Clone HI100; Cat. No. 304112) – flow cytometry<br>• Anti-CD62L: Biolegend (Clone DREG-56; Cat. No. 304816) – flow cytometry<br>• Anti-CD3: Biolegend (Clone HIT3a; Cat. No. 300309) – flow cytometry<br>• Anti-CD4: Biolegend (Clone A161A1; Cat. No. 357418) – flow cytometry<br>• Anti-CD8+a: BD Biosciences (Clone SK1; Cat. No. 563919, 612889, 345775, 570814)<br>• Anti-CD8+a: Biolegend (Clone SK1; Cat. No. 344710))<br>• Anti-CD8+a: Biolegend (Clone HIT8a; Cat. No. 300919)<br>• Anti-CD127: BD Biosciences (Clone HIL-7R-M21; Cat. No. 742547)<br>• Dynabeads human T-activator CD3/CD28: Gibco (Cat. No. 11132D)<br>• Anti-m^{6}A: Abcam (Cat. No. ab151230) – miCLIP<br>• Anti-human FTO: Abcam (Cat. No. ab92821) - Western Blot<br>• Anti-METTL3: proteintech (Cat. No. 15073-1-AP) – Western Blot<br>• Anti-human RhoGDI: Abnova (Cat. No. 89-113-917) - Western Blot<br>• CD8+ MicroBeads (human): MACS Miltenyi (Cat. No. 130-045-201)<br>• Total CD8+ T cell isolation kit (human): MACS Miltenyi (Cat. No. 130-096-495)
Validation	All antibodies were validated by the suppliers for the stated applications, with additional in-house validation where appropriate. In-house validation included:<br>• Immunoblotting: Detection of target proteins at expected molecular weights, with loss of signal upon CRISPR/Cas9-mediated knockout or inhibitor treatment where applicable.<br>• Flow cytometry: Staining patterns consistent with known CD8$^+$ T cell phenotypes; negative controls confirmed specificity.<br>• Loading controls: Stable RhoGDI expression across conditions.<br>All antibodies are listed in the Methods section with RRID to enable unambiguous reagent identification and reproducibility.

Eukaryotic cell lines

Policy information about cell lines and Sex and Gender in Research

Cell line source(s)	Francis Crick Institute - Cell Services
Authentication	None of the cell lines were authenticated.

Mycoplasma contamination	All cell lines tested negative for mycoplasma contamination.
Commonly misidentified lines (See ICLAC register)	No commonly misidentified cell lines were used in this study.

Plants

Seed stocks	n/a
Novel plant genotypes	n/a
Authentication	n/a

Flow Cytometry

Plots

Confirm that:

☒ The axis labels state the marker and fluorochrome used (e.g. CD4-FITC).

☒ The axis scales are clearly visible. Include numbers along axes only for bottom left plot of group (a 'group' is an analysis of identical markers).

☒ All plots are contour plots with outliers or pseudocolor plots.

☒ A numerical value for number of cells or percentage (with statistics) is provided.

Methodology

Sample preparation	The cells were pelleted by centrifugation wash with 1xPBS and stained with antibodies in FACS Buffer (5% FBS, 2 mM EDTA in PBS) at 4°C for 30–60 min. The stained cells were washed with FACS buffer 2–3 times and processed for flow cytometry.
Instrument	BD LSR-Fortessa, BD FACSymphony, Sony Spectra Analyser
Software	FlowJo (BD Biosciences, version 10)
Cell population abundance	Cell population abundance is shown in the gates of the figures. At least 10,000 events were recorded per sample. High CD8 - live positive cells were recorded for downstream analysis.
Gating strategy	Gating strategy: FSC-A/SSC-A -> FSC-H/FSC-A -> Live cells/SSC-A -> CD8positive cells OR CD8+/CD3+ ->other markers

☒ Tick this box to confirm that a figure exemplifying the gating strategy is provided in the Supplementary Information.
