# Supplementary material for: Meta-unstable mRNAs in activated CD8+ T cells are defined by interlinked AU-rich elements and m6A mRNA methylation
Source: Nat Commun. 2026 Jan 22;17:160. doi: 10.1038/s41467-025-67762-w (PMC12827480; doi:10.1038/s41467-025-67762-w)

Figure S1C – uncropped (1)

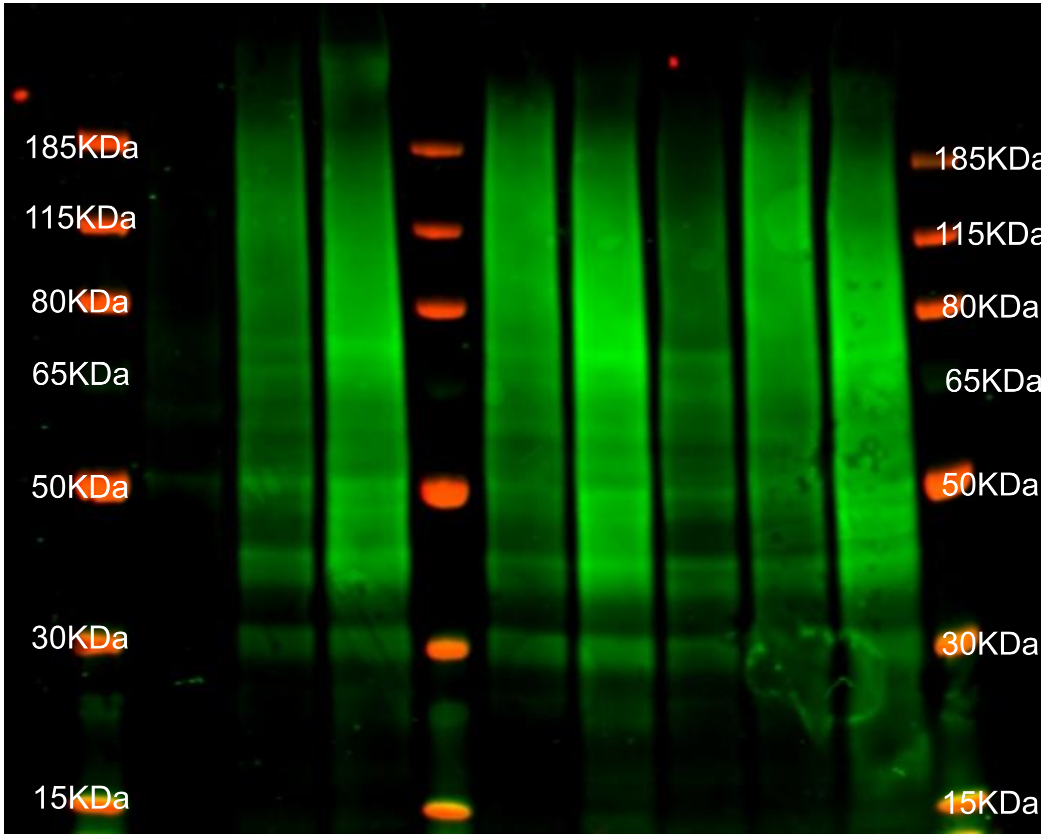

Figure S1C – uncropped (2)

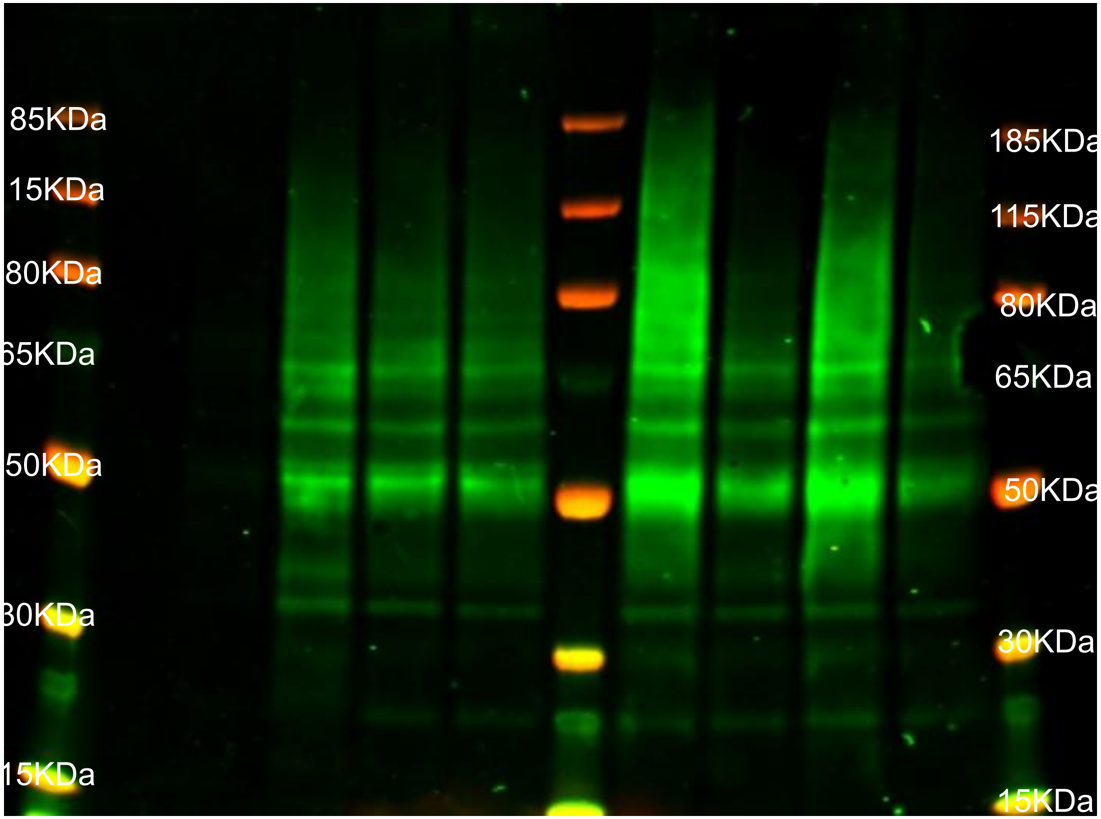

Figure S1C – uncropped (3)

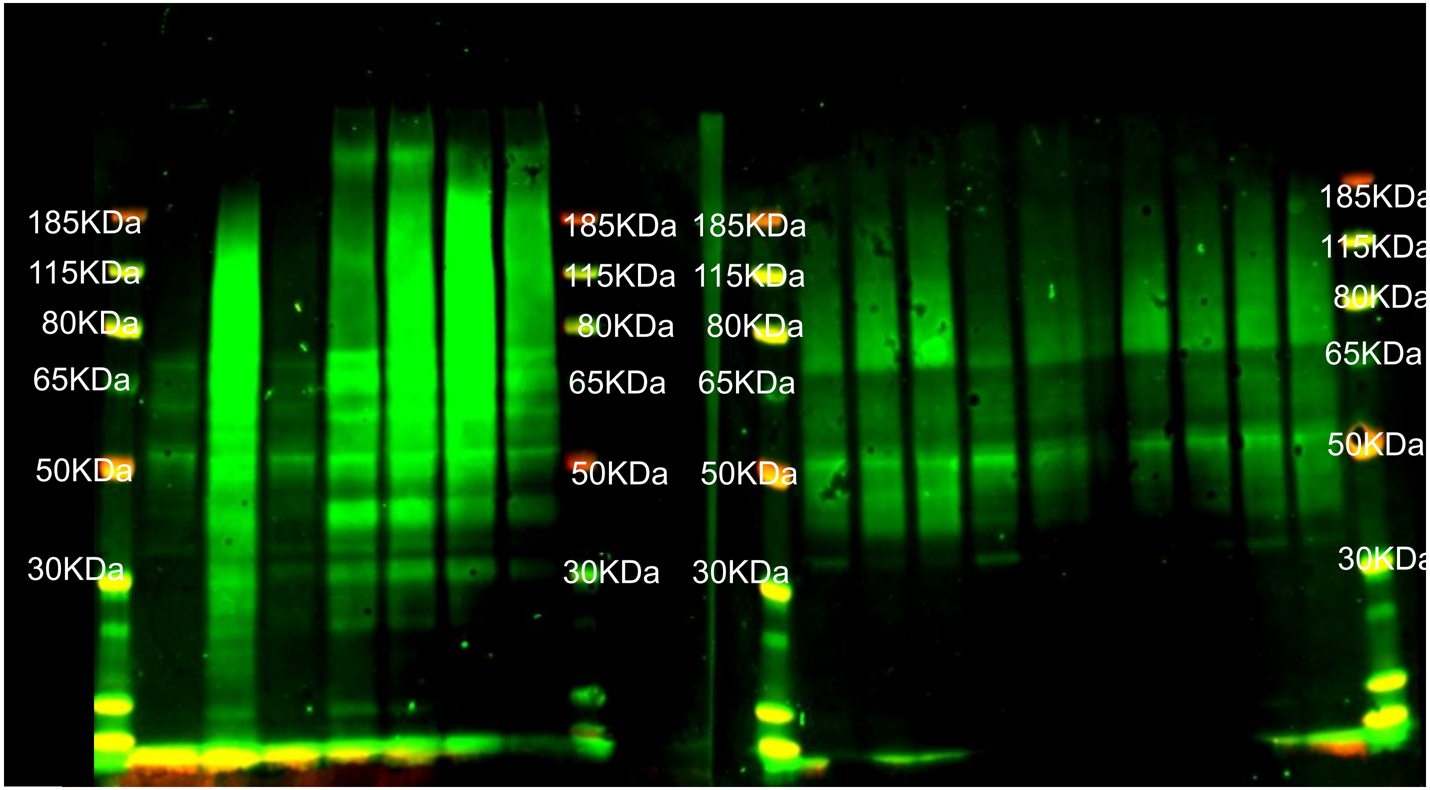

Supplement: Supplementary file 6 — Source Data [file 41467_2025_67762_MOESM6_ESM.zip › Source Data/Fig-S1C.pdf]
