# Supplementary figures and images for: Meta-unstable mRNAs in activated CD8+ T cells are defined by interlinked AU-rich elements and m6A mRNA methylation
Source: Nat Commun. 2026 Jan 22;17:160. doi: 10.1038/s41467-025-67762-w (PMC12827480; doi:10.1038/s41467-025-67762-w)

Panel S3B

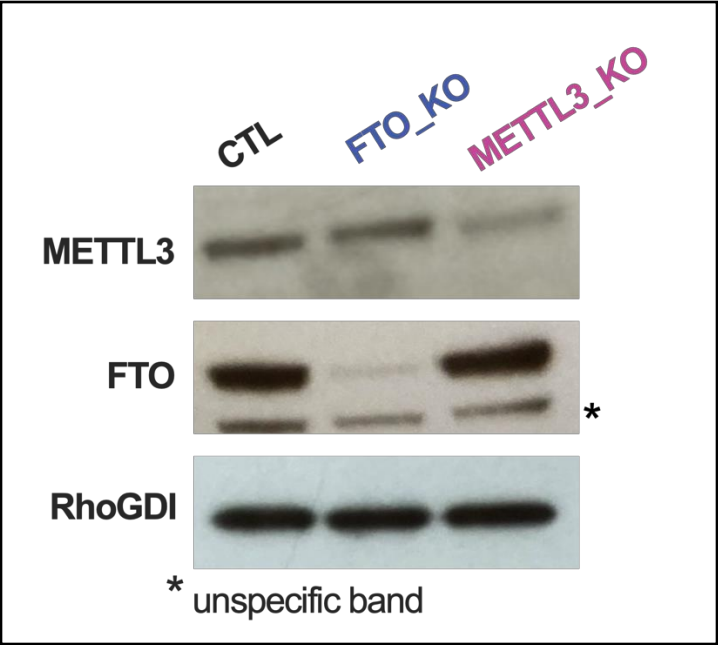

Uncropped images

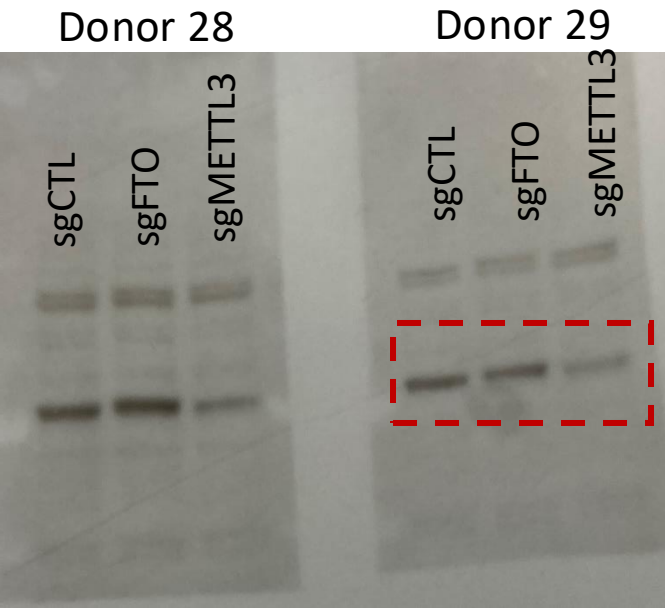

METTL3 - proteintech (Cat. No. 15073-1-AP)

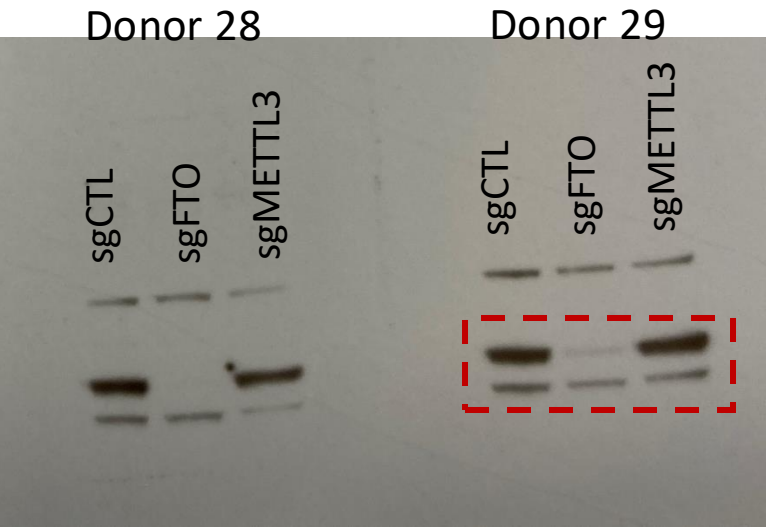

FTO - Abcam (Cat. No. ab92821)

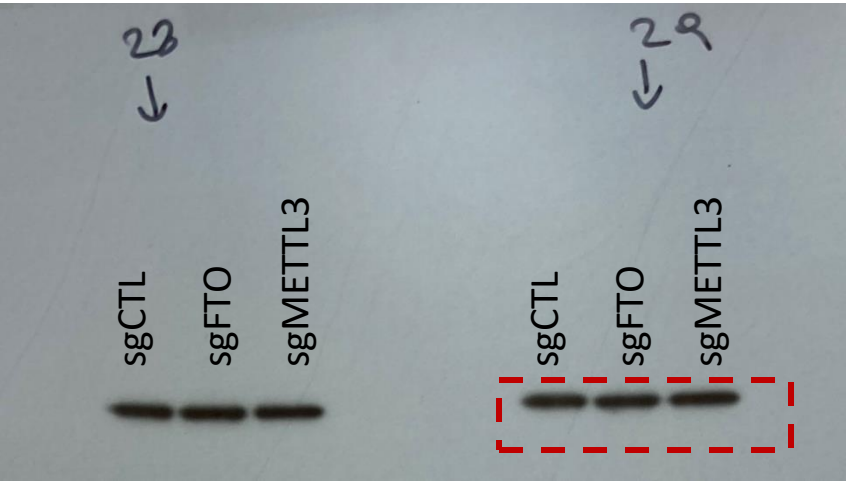

RhoGDI - Abnova (Cat. No. 89-113-917)

Supplement: Supplementary file 6 — Source Data [file 41467_2025_67762_MOESM6_ESM.zip › Source Data/Fig-S3B.pdf]

Figure S4A – uncropped

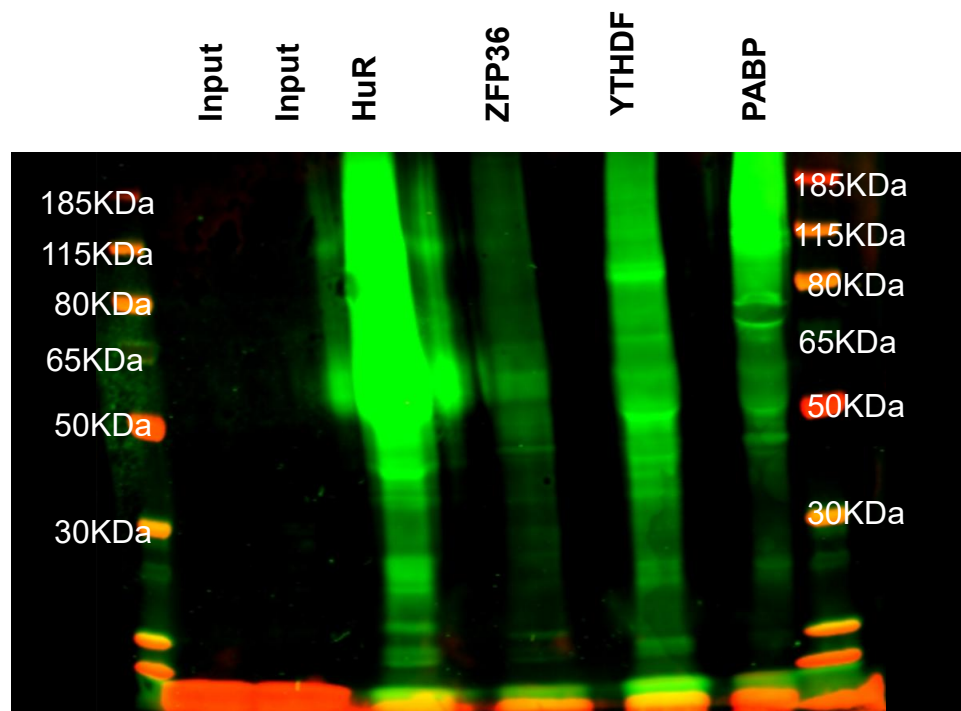

Figure S4A – membrane cut for LC-MS

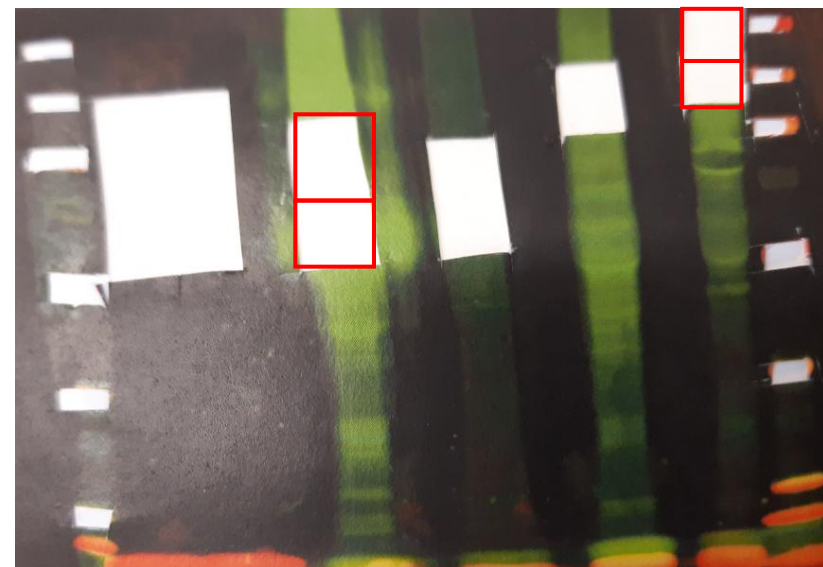

Supplement: Supplementary file 6 — Source Data [file 41467_2025_67762_MOESM6_ESM.zip › Source Data/Fig-S4A.pdf]
